# Dynamics of Serum Cytokines and Chemokines in Patients With Idiopathic Multicentric Castleman Disease: From a Phase Ib Investigator-Initiated Trial of Filgotinib

**DOI:** 10.7759/cureus.78974

**Published:** 2025-02-13

**Authors:** Shoichi Fukui, Remi Sumiyoshi, Tomohiro Koga, Naoki Hosogaya, Sawana Narita, Shimpei Morimoto, Hiroshi Yano, Atsushi Katsube, Shingo Yano, Yasufumi Masaki, Shinichiro Tsunoda, Shuzo Sato, Kiyoshi Migita, Atsushi Kawakami

**Affiliations:** 1 Department of Immunology and Rheumatology, Division of Advanced Preventive Medical Sciences, Nagasaki University Graduate School of Biomedical Sciences, Nagasaki, JPN; 2 Clinical Research Center, Nagasaki University Hospital, Nagasaki, JPN; 3 Division of Clinical Oncology/Hematology, Department of Internal Medicine, The Jikei University School of Medicine, Tokyo, JPN; 4 Department of Hematology and Immunology, Kanazawa Medical University, Uchinada, JPN; 5 Divison of Clinical Immunology and Hematology, Department of Internal Medicine, Sumitomo Hospital, Osaka, JPN; 6 Department of Rheumatology, Fukushima Medical University School of Medicine, Fukushima, JPN

**Keywords:** fibroblast growth factor (fgf-2), il-4, interleukin (il)-6, tnf-β, vegf-a

## Abstract

Background

Idiopathic multicentric Castleman disease (iMCD) is a chronic inflammatory condition for which Janus kinase (JAK) inhibition has been hypothesized to be a potential treatment. However, filgotinib, a JAK1 preferential inhibitor, did not show apparent efficacy for iMCD in a clinical trial at eight weeks. This study aimed to compare the serum cytokine and chemokine profiles of patients treated with filgotinib with those of patients treated with tocilizumab to speculate why filgotinib was not effective at eight weeks.

Methods

This study included five patients treated with filgotinib who participated in a phase Ib single-arm clinical trial of filgotinib for iMCD and five tocilizumab-treated patients whose data were collected retrospectively. Serum levels of 41 cytokines/chemokines before and after treatment were measured.

Results

The tocilizumab group showed improvement in C-reactive protein, hemoglobin, and albumin levels after treatment while the filgotinib group showed no changes in these markers. The tocilizumab group showed significant changes in 12 cytokines/chemokines from baseline to after treatment, whereas the filgotinib group showed only a decrease in IL-18 and IL-27 levels. After treatment, significant differences were observed between the two groups for 10 cytokines/chemokines. Five cytokines (FGF-2, IL-4, IL-6, TNF-β, and VEGF-A) showed significant changes after tocilizumab treatment and differences between the tocilizumab and filgotinib groups after treatment.

Conclusion

This study identified FGF-2, IL-4, IL-6, TNF-β, and VEGF-A as potential factors that could explain the lack of apparent efficacy of filgotinib in iMCD treatment at eight weeks. These findings may contribute to future drug development for iMCD.

## Introduction

Idiopathic multicentric Castleman disease (iMCD) is a rare disease characterized by systemic lymphadenopathy with constitutional symptoms (fever, night sweats, weight loss, and fatigue) [1]. Research has shown that elevated interleukin-6 (IL-6) levels correlate with clinical symptoms, leading to the development of the IL-6 inhibitors, tocilizumab [2] and siltuximab [3] for iMCD. These treatments are effective for iMCD; however, some patients experience intractable disease. Although various chemotherapeutic agents have been tried for refractory cases [4], evidence supporting their use remains minimal.

Recent research has focused on Janus kinases (JAKs), which regulate downstream of cytokine receptors, including IL-6 [5]. JAKs contribute to the signal transducer and activator of transcription 3 activation in iMCD pathogenesis [6], suggesting that JAK-STAT signaling inhibition is a potential treatment approach. This rationale led to a phase Ib investigator-led clinical trial of filgotinib for iMCD that was conducted at five facilities across Japan in 2024. Filgotinib, which has been approved for use in rheumatoid arthritis (RA) [7] and ulcerative colitis [8], is a JAK1 preferential inhibitor that suppresses downstream signals of IL-6, and it was anticipated that filgotinib would also be effective in iMCD. However, the treatment response at eight weeks was not promising because C-reactive protein (CRP), hemoglobin, and albumin levels showed no changes [9]. Further research is needed to understand why filgotinib did not show apparent efficacy for the future development of treatments for iMCD.

In this study, we aimed to elucidate the changes in serum cytokines and chemokines in patients treated with filgotinib by comparing them with those in patients treated with tocilizumab, the standard treatment for iMCD. This study was an exploratory post-hoc analysis using preserved sera from a clinical trial, and sera collected separately from the clinical trial. We anticipated that understanding the dynamics of multiple cytokines and chemokines would provide speculation on the cause of the lack of apparent efficacy of filgotinib in iMCD.

## Materials and methods

Study design and patients

This study used sera from iMCD patients who participated in a single-arm trial of filgotinib (200 mg daily) registered in the Japan Registry of Clinical Trials (https://jrct.niph.go.jp/) as jRCT2071230108 (approved by the Nagasaki University Hospital Institutional Review Board, approval no. 123-002). The study was conducted from December 2023 to June 2024. In addition, sera from patients treated with tocilizumab were collected to be compared to those from patients treated with filgotinib. Patients in the tocilizumab group were diagnosed with iMCD at Nagasaki University Hospital between September 2018 and October 2023. Sera at baseline and eight weeks (filgotinib) or later (tocilizumab, median 28 months (minimum 11 - maximum 73 months)) were used. Clinical information of patients treated with tocilizumab, referring to clinical records, was collected retrospectively.

Criteria

All patients were diagnosed with iMCD according to the diagnostic criteria issued by Japan's Ministry of Health, Labour and Welfare, i.e., “Designated intractable diseases that became effective on April 1, 2018 (notice no. 331).” Patients with a total score on the CHAP (C-reactive protein (CRP), Hemoglobin, Albumin, + Performance Status (PS, Eastern Cooperative Oncology Group (ECOG))) [10] that was ≥2 points in total with hemoglobin or albumin ≥1 point and CRP ≥1 point at baseline. Patients with iMCD-TAFRO (thrombocytopenia, anasarca, fever, reticulin fibrosis, renal insufficiency, and organomegaly clinical subtype) as defined by the validated international definition [11] were excluded.

Outcomes

Endpoints were as follows at eight weeks of treatment (filgotinib) or later (tocilizumab): (1) the patient's CHAP score [10], (2) CRP (mg/dL), (3) hemoglobin (g/dL), (4) albumin (g/dL), and (5) the ECOG-PS.

Multiple cytokines/chemokines measurements

The sera of the patients were analyzed using the Milliplex^®^ MAP Human Cytokine/Chemokine Magnetic Bead Panel-Premixed 41 Plex panel (Merck Millipore, Billerica, MA, USA) and MAGPIX® with xPONENT^®^ software (Luminex Corp., Austin, TX, USA). The levels of 41 cytokines/chemokines were measured as follows: epidermal growth factor (EGF), CCL11/eotaxin, basic fibroblast growth factor (FGF-2/bFGF), FMS-like tyrosine kinase 3 ligand (FLT-3 L), fractalkine, granulocyte colony-stimulating factor (G-CSF), granulocyte-macrophage colony-stimulating factor (GM-CSF), chemokine (C-X-C motif) ligand 1 (CXCL1/GRO-α), interferon (IFN)-α2, IFN-γ, interleukin (IL)-10, IL-12p40, IL-12p70, IL-13, IL-15, IL-17A, IL-17F, IL-18, IL-1 receptor antagonist (IL-1RA), IL-1α, IL-1β, IL-2, IL-22, IL-27, IL-4, IL-5, IL-6, IL-7, IL-8, C-X-C motif chemokine ligand 10 (CXCL10/IP-10), monocyte chemoattractant protein-1 (MCP-1/CCL2), MCP-3, macrophage-derived chemokine (MDC), CCL3/macrophage inflammatory protein (MIP)-1α, CCL4/MIP-1β, platelet-derived growth factor (PDGF)-AA, transforming growth factor (TGF)-α, tumor necrosis factor (TNF)-α, TNF-β, vascular endothelial growth factor (VEGF)-A, and soluble CD40 ligand (sCD40L).

Healthy donors

Sera from healthy donors - residents of the town of Saza in Nagasaki prefecture - who underwent specific health checkups in 2016 were used for the 41 Plex panel as the controls to calculate the normal limits with 95% confidence interval (approved by the Ethics Committee of the Nagasaki University Graduate School of Biomedical Sciences, project registration number: 14051404). All healthy donors had no past or present medical history of inflammatory disease (n=101 (59 women), mean age 58 (standard deviation: 10) years).

Ethics

This study complied with the Declaration of Helsinki and was approved by the Nagasaki University Hospital Institutional Review Board (approval no. 24120503). An opt-out approach was used to obtain patient consent for the study.

Statistical analyses

Categorical and continuous variables are described as frequencies and means with standard deviations unless otherwise specified. Associations between variables were assessed using the student's t-test for continuous variables. The paired t-test was used to assess differences in serum cytokine and chemokine levels at baseline and after treatment. All hypothesis tests were conducted at a significance level of 0.05. The selective process of null hypotheses or multiplicity of the tests was not considered because of the exploratory objective of the analyses [12]. All statistical analyses were performed using R ver. 4.3.2 (R Foundation for Statistical Computing, Vienna, Austria).

## Results

Patient characteristics at baseline

Table 1 presents the baseline characteristics. The median patient age was 62 and 60 years in the tocilizumab and filgotinib groups, respectively. The lymph node histology at diagnosis was plasma cell type, and neither the hyaline-vascular type nor the mixed type was observed in either group. All patients were diagnosed with idiopathic plasmacytic lymphadenopathy (IPL) based on the criteria for IPL [13], which was modified from the original criteria by Mori et al. [14]. The median CRP levels were 4.90 mg/dL and 6.73 mg/dL, respectively. The median CHAP (CRP, Hemoglobin, Albumin, + PS (ECOG)) score at baseline was five in both groups.

**Table 1:** Characteristics of patients with idiopathic multicentric Castleman disease treated with either tocilizumab or with filgotinib at baseline and after treatment Abbreviation: CHAP: CRP, hemoglobin, albumin, + PS (ECOG), CRP: C-reactive protein, ECOG-PS: Eastern Cooperative Oncology Group-Performance Status, VAS: visual analog scale. *for the filgotinib group, **for the tocilizumab group

Characteristics	Tocilizumab (n=5)	Filgotinib (n=5)
At baseline		
Age, years, median (min−max)	62 (43-69)	60 (37–61)
Sex, female, n (%)	2 (40)	2 (40)
Height, cm, median (min−max)	160 (152-172)	170 (152–176)
Body weight, kg, median (min−max)	55.7 (46.1-72.5)	73.7 (50.9–75.0)
Treatment-naïve, n (%)	5 (100)	3 (60)
Previous treatment with immunosuppressant, n (%)		
Prednisolone	0 (0)	2 (40)
Others	0 (0)	0 (0)
Concomitant use of immunosuppressant, n (%)		
Prednisolone 10 mg/day	0 (0)	1 (20)
Others	0 (0)	0 (0)
Histology, n (%)		
Hyaline vascular type	0 (0)	0 (0)
Plasma cell type	5 (100)	5 (100)
Mixed type	0 (0)	0 (0)
CRP, mg/dL, median (min−max)	4.90 (3.70-7.22)	6.73 (2.47–7.45)
Hemoglobin, g/dL, median (min−max)	9.3 (8.5-11.6)	10.1 (8.1–11.8)
Albumin, g/dL, median (min−max)	2.4 (2.4-2.7)	2.9 (2.5–3.3)
ECOG-PS, n (%)		
0	4 (80)	2 (40)
1	1 (20)	3 (60)
CHAP score, median (min−max)	5 (3-7)	5 (3–6)
Platelet count, ×10^3^/μL, median (min−max)	338 (262-456)	339 (226-556)
Immunoglobulin G, mg/dL, median (min−max)	4,436 (3,733-4,996)	5,357 (4,446-5,968)
At 8 weeks* or later**		
CRP, mg/dL, median (min−max)	0.01 (0.01-0.04)	6.13 (1.88-6.82)
Hemoglobin, g/dL, median (min−max)	14.1 (12.6-14.3)	10.0 (8.0-12.4)
Albumin, g/dL, median (min−max)	4.2 (4.0-4.3)	3.1 (2.7-3.4)
ECOG-PS, n (%)		
0	5 (100)	4 (80)
1	0 (0)	1 (20)
CHAP score, median (min−max)	0 (0-0)	3 (2-5)

Outcomes at eight weeks or later

All five patients in the filgotinib group completed eight weeks of treatment with no discontinuation. The median CRP levels after treatment were 0.01 mg/dL (tocilizumab) and 6.13 mg/dL (filgotinib), respectively. The median hemoglobin level and albumin level after treatment were 14.1 g/dL and 10.0 g/dL, and 4.2 g/dL and 3.1 g/dL, respectively. The median CHAP scores were 0 and 3, respectively.

Comparison of cytokines and chemokines between the two groups

Table 2 shows the serum levels of cytokines and chemokines in both groups.

**Table 2:** Serum levels of cytokines and chemokines at baseline and after treatment with the reference ranges of healthy donors Data are presented as mean (standard deviation). All units are pg/mL. Variables were compared between groups using the Student's t-test (paired t-test for within-group comparisons). T-statistics and the corresponding p-values are shown for ^1)^tocilizumab at baseline vs. tocilizumab after treatment, ^2)^filgotinib at baseline vs. filgotinib after treatment by paired t-test, and ^3)^tocilizumab vs. filgotinib after treatment. CI: confidence interval

	Tocilizumab at baseline (n=5)	Filgotinib at baseline (n=5)	Tocilizumab after treatment (n=5)	Filgotinib at 8 weeks (n=5)	95% CI of healthy donors (n=101)	^1^^)^t-statistic	^1)^p-value	^2)^t-statistic	^2)^p-value	^3)^t-statistic	^3)^p-value
EGF	177 (139)	51 (26)	160 (105)	64 (46)	353-451	0.75	0.494	-1.18	0.304	1.89	0.112
Eotaxin	113 (57)	95 (43)	172 (88)	131 (51)	114-138	-2.56	0.063	-1.20	0.295	0.88	0.409
FGF-2	234 (180)	331 (186)	94 (73)	327 (166)	32-66	2.86	0.046	0.11	0.915	-2.87	0.031
FLT-3L	14 (7)	13 (11)	10 (5)	12 (6)	36-54	2.92	0.043	0.30	0.782	-0.67	0.525
Fractalkine	546 (654)	278 (334)	143 (65)	236 (227)	109-145	1.52	0.204	0.85	0.445	-0.87	0.425
G-CSF	38 (19)	58 (33)	38 (6)	80 (79)	2-22	-0.03	0.978	-0.94	0.401	-1.18	0.303
GM-CSF	68 (91)	35 (64)	7 (15)	44 (55)	3-7	1.51	0.206	-0.40	0.707	-1.46	0.209
GRO	75 (42)	46 (25)	29 (10)	49 (30)	16-24	2.75	0.052	-0.70	0.521	-1.43	0.213
IFNα2	233 (287)	164 (256)	39 (71)	145 (207)	15-49	1.84	0.139	0.65	0.549	-1.09	0.328
IFNγ	204 (81)	134 (30)	121 (42)	130 (24)	2-8	2.71	0.053	0.43	0.689	-0.41	0.698
IL-10	9 (5)	22 (27)	5 (2)	11 (8)	0-8	1.56	0.194	1.13	0.323	-1.77	0.142
IL-12p40	117 (53)	71 (35)	64 (15)	56 (32)	24-36	1.81	0.145	2.20	0.093	0.50	0.636
IL-12p70	27 (49)	85 (177)	2 (1)	79 (165)	0-0	1.14	0.317	1.05	0.354	-1.05	0.354
IL-13	200 (231)	136 (97)	47 (31)	124 (71)	45-79	1.69	0.167	0.93	0.404	-2.22	0.072
IL-15	17 (10)	29 (16)	14 (8)	30 (14)	9-17	1.80	0.147	-0.18	0.863	-2.21	0.066
IL-17A	30 (30)	26 (20)	8 (7)	27 (22)	3-13	1.88	0.133	-0.55	0.613	-1.82	0.132
IL-17F	11 (14)	28 (23)	6 (12)	25 (19)	2-16	3.31	0.030	0.47	0.661	-1.90	0.100
IL-18	21 (34)	29 (9)	11 (13)	17 (6)	15-21	1.02	0.367	3.49	0.025	-0.91	0.401
IL-1ra	8 (3)	8 (2)	4 (1)	8 (4)	1-7	2.35	0.078	0.15	0.887	-2.14	0.085
IL-1α	21 (19)	34 (26)	3 (2)	34 (33)	13-37	2.33	0.080	0.01	0.991	-2.08	0.105
IL-1β	19 (13)	29 (12)	9 (4)	28 (12)	12-36	2.57	0.062	0.05	0.965	-3.54	0.016
IL-2	2 (2)	3 (2)	0 (1)	4 (2)	0-2	1.78	0.150	-0.52	0.628	-3.69	0.016
IL-22	380 (224)	296 (198)	123 (95)	289 (142)	1-13	3.08	0.037	0.22	0.839	-2.18	0.066
IL-27	1930 (1470)	1204 (299)	1193 (662)	978 (348)	1496-1882	1.98	0.119	2.79	0.049	0.64	0.545
IL-4	5 (3)	9 (10)	2 (1)	4 (1)	0-2	3.49	0.025	1.39	0.237	-3.39	0.013
IL-5	3 (1)	3 (3)	3 (2)	3 (2)	6-10	0.11	0.918	-0.44	0.686	-0.75	0.479
IL-6	26 (15)	16 (6)	295 (217)	14 (4)	2-2	-2.84	0.047	1.43	0.226	2.90	0.044
IL-7	12 (3)	47 (78)	8 (5)	33 (49)	6-24	2.27	0.086	1.09	0.336	-1.13	0.319
IL-8	169 (262)	13 (2)	11 (5)	14 (4)	86-184	1.33	0.256	-0.48	0.657	-0.85	0.420
IP-10	189 (100)	105 (62)	248 (82)	68 (38)	124-158	-1.72	0.161	2.03	0.112	4.42	0.005
MCP-1	268 (95)	295 (127)	510 (172)	280 (167)	567-661	-3.35	0.029	0.53	0.627	2.15	0.064
MCP-3	115 (50)	58 (18)	37 (11)	54 (24)	14-22	4.24	0.013	1.46	0.218	-1.42	0.208
MDC	375 (58)	384 (120)	381 (39)	362 (128)	691-815	-0.19	0.860	2.17	0.096	0.31	0.767
MIP-1α	38 (16)	36 (9)	24 (7)	38 (9)	31-41	1.80	0.147	-0.47	0.661	-2.63	0.032
MIP-1β	51 (20)	38 (11)	40 (18)	36 (7)	47-61	0.89	0.426	0.42	0.699	0.43	0.683
PDGF-AA	13479 (5020)	7649 (3578)	9433 (3821)	7809 (3757)	4754-5588	4.46	0.011	-0.37	0.732	0.68	0.517
TGFα	11 (3)	13 (11)	6 (5)	11 (8)	5-9	3.20	0.033	0.96	0.390	-1.11	0.308
TNFα	65 (36)	53 (20)	41 (11)	43 (23)	14-18	1.50	0.207	2.31	0.082	-0.20	0.846
TNFβ	84 (56)	44 (26)	17 (9)	40 (19)	5-9	2.98	0.041	0.91	0.414	-2.47	0.049
VEGF-A	701 (417)	634 (231)	206 (135)	501 (161)	279-371	3.61	0.023	2.04	0.111	-3.13	0.014
sCD40L	6050 (1989)	1607 (991)	6473 (3474)	1824 (741)	2223-2959	-0.25	0.815	-0.41	0.701	2.93	0.039

**Figure 1:**
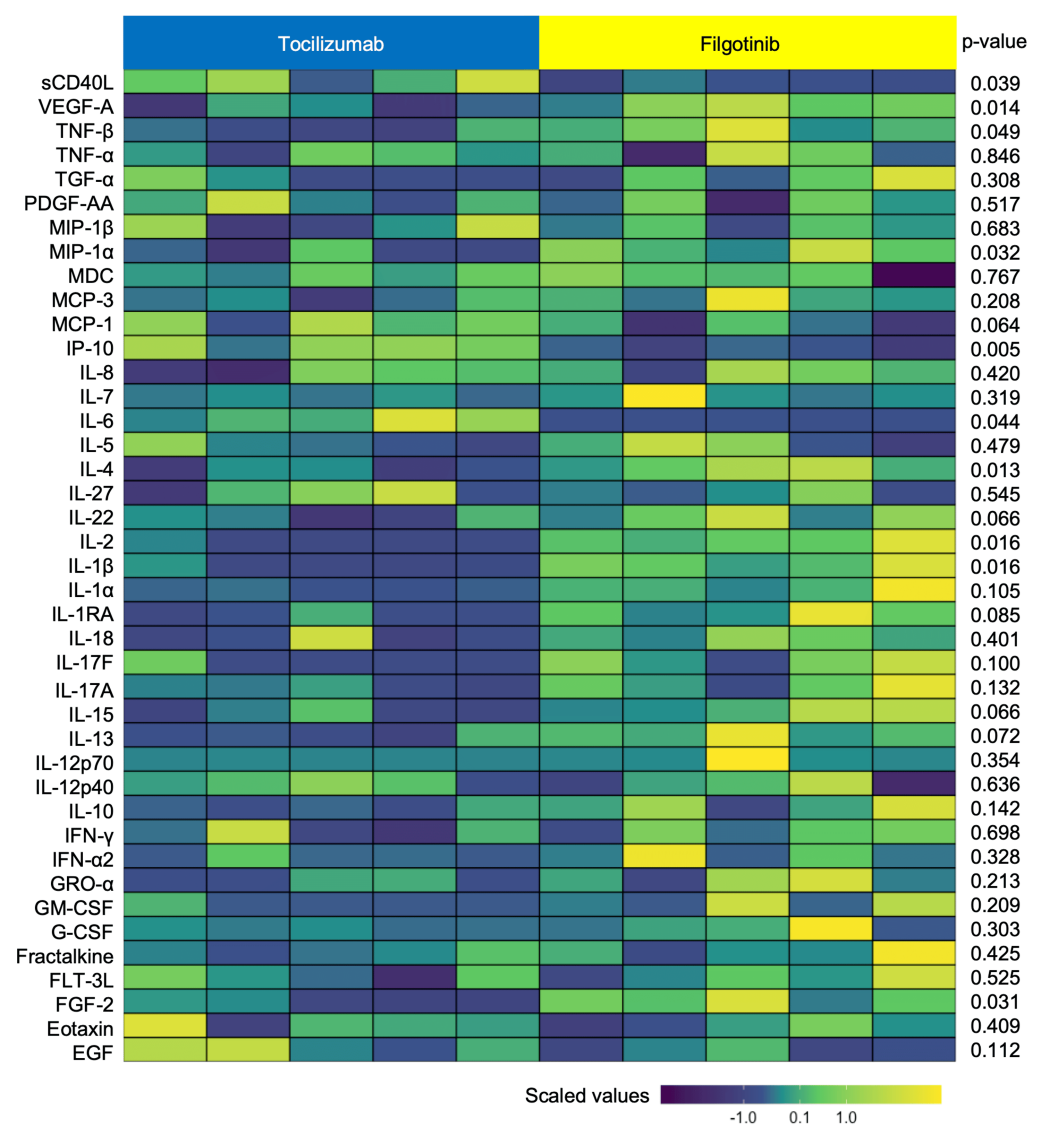
Heat map of serum cytokines and chemokines after treatment of the tocilizumab group and the filgotinib group The p-values were calculated using the student’s t-test to compare cytokines and chemokines between the two groups. Colors represent scaled values of cytokines and chemokines.

The tocilizumab group showed a significant decrease in the serum levels of FGF-2, FLT-3L, IL-17F, IL-22, IL-4, MCP-3, PDGF-AA, TGF-α, TNF-β, and VEGF-A, and a significant increase in the serum levels of IL-6 and MCP-1 from baseline to after treatment. The filgotinib group showed a significant decrease in serum levels of IL-18 and IL-27 from baseline to after treatment. Figure 1 shows the differences in serum cytokines and chemokines after treatment between the two groups on a heat map. Significant differences were observed in the serum levels of FGF-2, IL-1β, IL-2, IL-4, IL-6, IP-10, MIP-1α, TNF-β, VEGF-A, and sCD40L between the tocilizumab and filgotinib groups after treatment.

**Figure 2:**
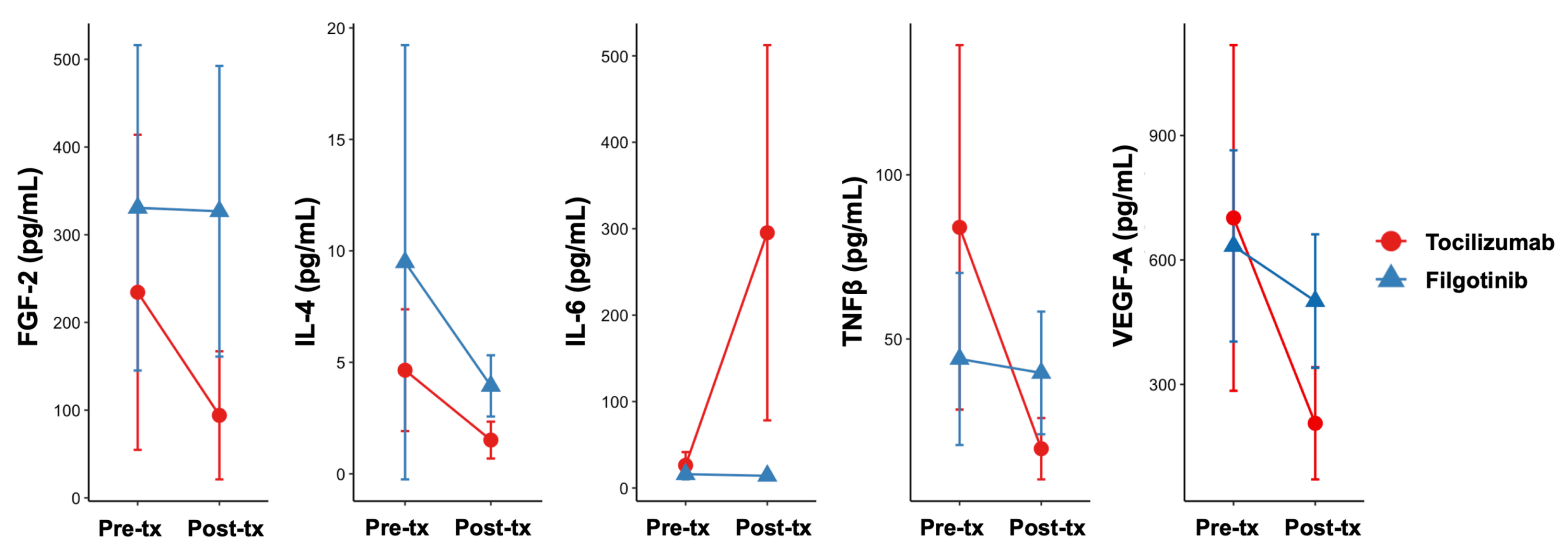
Cytokines with significant differences between the two groups Pre-tx: pre-treatment, Post-tx: post-treatment

Serum cytokines and chemokines that were significantly different between at baseline and after treatment in the tocilizumab group and significantly different between tocilizumab and filgotinib after treatment included FGF-2, IL-4, IL-6, TNF-β, and VEGF-A (Figure 2). All cytokines were lower in the tocilizumab group than in the filgotinib group after treatment, except for IL-6.

## Discussion

This exploratory study showed significant differences in the serum levels of FGF-2, IL-4, IL-6, TNF-β, and VEGF-A between the tocilizumab and filgotinib groups after treatment among cytokines and chemokines that significantly changed after tocilizumab administration. Here, we summarize and discuss previous reports on the relationship between these cytokines and iMCD or Castleman disease.

TNF-β, also known as lymphotoxin-α, plays a role in regulating the growth and function of lymphocytes. Elevated levels of TNF-β mRNA have been observed in lymph nodes in Castleman disease [15]. In addition, serum proteomic analyses have identified TNF signaling as a highly enriched pathway in iMCD patients [16], which is not limited to TNF-β. Elevated serum TNF-β levels in iMCD above the upper limit of the normal range from healthy donors may contribute to the inflammatory state and lymph node abnormalities of iMCD.

VEGF stimulates angiogenesis. Elevated VEGF levels were observed in the sera and lymph node supernatants of patients with Castleman disease compared with those of normal controls [17,18]. In addition, immunohistochemical analyses have revealed VEGF expression in plasma cells within the interfollicular regions of affected lymph nodes [17,18]. VEGF expression was also detected in the germinal center of Castleman disease cases, unlike in the normal tonsillar germinal center [19]. Based on these reports, VEGF is thought to contribute to the pathophysiology of iMCD.

Elevation of IL-6 levels after the initiation of tocilizumab in Castleman disease has been reported [20]. Therefore, our results of elevated serum IL-6 levels after tocilizumab treatment are consistent with a previous report. Regarding no changes in IL-6 in the filgotinib group, we concluded that filgotinib could not inhibit IL-6 at eight weeks in this study because a case report of iMCD-not otherwise specified (NOS) mentioned the time-dependent decrease of serum IL-6 by ruxolitinib with dexamethasone [21]. However, it should be noted that the transcriptomic levels of cytokines differ between iMCD-NOS/TAFRO and iMCD-IPL in lymph nodes [22], which may affect serum cytokines because our study included only patients with iMCD-IPL.

In RA, filgotinib decreased the serum levels of multiple cytokines, Th1-related cytokines (IL-2, IFN-γ, and IL-12), Th2-related cytokines (IL-4, IL-5, and IL-13), and Th17-related cytokines (IL-1β, IL-6, IL-17A, IL-21, and IL-23) at 12 weeks [23]. Another study in RA reported that filgotinib decreased ﻿CRP levels by 77.4% and IL-6 levels by 13.6% relative to placebo at 12 weeks [24]. Based on these reports, filgotinib in iMCD did not seem to suppress any cytokines at eight weeks, although attention is required for the short duration of treatment with filgotinib.

To the best of our knowledge, FGF-2 and IL-4 have not been reported to be associated with iMCD pathogenesis. The contributions of fibroblasts and Th2 cells to the pathogenesis of iMCD will be considered in the future.

This study has several limitations. First, this study compared only five patients in the tocilizumab group and five patients in the filgotinib group because the filgotinib trial recruited only five patients. The small sample size limits statistical power. Second, the evaluation at eight weeks in the filgotinib group may have been too short to evaluate the effects of filgotinib on serum cytokines and chemokines. The small sample size and short duration of treatment in the filgotinib group limit the generalizability of our findings. Third, the timing of serum collection after treatment initiation varied in the tocilizumab group because the tocilizumab group was retrospectively collected. Additionally, the retrospective nature of the tocilizumab group introduced a potential selection bias. Fourth, because none of the patients in the filgotinib group showed apparent efficacy, it was impossible to distinguish whether the differences in cytokines and chemokines between the tocilizumab and filgotinib groups were due to the difference in the effects and durations of treatment or the difference in disease activity after treatment. Fifth, the statistical significance found in this study was based on the p-values that presumed the values were not greater than the nominal type-1 error rate, that is, the usual definition of the p-value [12]. Therefore, the results lack statistical validity.

This study has its strengths. We have provided data on the time course of multiple cytokines and chemokines in iMCD, a rare disease. We focused on FGF-2, IL-4, IL-6, TNF-β, and VEGF-A based on the statistical analysis in this report; however, other intriguing findings in the data may be helpful for other clinicians and researchers. Despite the above-mentioned limitations, our speculative findings may suggest why filgotinib did not show apparent clinical efficacy.

## Conclusions

In this exploratory analysis comparing the treatment of iMCD with tocilizumab and filgotinib, we identified significant differences in several key cytokines, which may explain the lack of apparent efficacy of filgotinib. Most notably, FGF-2, IL-4, IL-6, TNF-β, and VEGF-A levels showed distinct patterns. The persistent elevation of TNF-β and VEGF-A levels in patients treated with filgotinib suggests that JAK1-preferential inhibition may be insufficient to control the complex inflammatory cascade in iMCD. While our findings are limited by the small sample size and short duration of filgotinib treatment, these results not only help explain the outcomes of current standard treatment but also provide potential implications for the future development of effective treatments for patients with iMCD.
